# Review of icatibant use in the Winnipeg Regional Health Authority

**DOI:** 10.1186/s13223-020-00493-3

**Published:** 2020-11-11

**Authors:** George Cai, Colin Barber, Chrystyna Kalicinsky

**Affiliations:** 1 University of Manitoba, Winnipeg, Canada; 2 Section of Allergy and Clinical Immunology, GC319 Health Sciences Centre, 820 Sherbrook Street, Winnipeg, MB R3A 1R9 Canada

**Keywords:** Icatibant, Angioedema, ACE-inhibitor, Angiotensin-converting enzyme inhibitor, Hereditary angioedema

## Abstract

**Background:**

This is a retrospective review of the Winnipeg Regional Health Authority’s (WRHA) angioedema patients who were dispensed icatibant in hospital. Icatibant is a bradykinin B2 receptor antagonist indicated for Hereditary Angioedema (HAE) types I and II and is used off-label for HAE with normal C1INH (HAE-nC1INH) and ACE-inhibitor induced angioedema (ACEIIAE). The WRHA’s use of icatibant is regulated by the Allergist on call. We characterized icatibant's use and the timeline from patient presentation, compared the real-world experience with the FAST-3 trial and hypothesized the factors which may affect response to icatibant.

**Methods:**

Background data were collected on patients. Angioedema attack-related data included administered medications, performed investigations and the timeline to endpoints such as onset of symptom relief. Data was analyzed in R with the package “survival.” Time-to-event data was analyzed using the Peto–Peto Prentice method or Mann–Whitney U-test. Data was also compared with published clinical trial data using the Sign Test. Fisher’s Exact Test was used to produce descriptive statistics.

**Results:**

Overall, 21 patients accounted for 23 angioedema attacks treated with icatibant. Approximately half the patients had a diagnosis of HAE-nC1IHN and half of ACEIIAE. Of those presenting with angioedema, 65% were first treated with conventional medication. Patients without a prior angioedema diagnosis were evaluated only 40–50% of the time for C4 levels or C1INH function or level. The median time from patients’ arrival to the emergency department until the Allergy consultant’s response was 1.77 h. Patients with HAE-nC1IHN had median times to onset of symptom relief and final clinical outcome (1.13 h, p = 0.34; 3.50 h, p = 0.11) similar to those reported in FAST-3 for HAE I/II. Patients with ACEIIAE had longer median times to onset of symptom relief (4.86 h, p = 0.01) than predicted.

**Conclusions:**

HAE-nC1INH may be an appropriate indication for treatment with icatibant. Conversely, the results of this study do not support the use of icatibant for the treatment of ACEIIAE, concordant with a growing body of literature. Patients should be stratified into groups of more- or less-likely icatibant-responders through history and laboratory investigations in order to prevent potential delays.

## Introduction and background

Icatibant is a selective bradykinin B2 receptor antagonist that is used to treat bradykinin-mediated angioedema. It has been evaluated for use in types I and II hereditary angioedema (HAE) and angiotensin-converting enzyme inhibitor induced angioedema (ACEIIAE) in randomized control trials (RCTs) [1–6]. Overall, angioedema represents a significant burden to emergency departments (EDs) in North America: almost 300,000 ED visits occurred in the Winnipeg Regional Health Authority (WRHA) from 2016 to 2017 and 1 in 1000 ED visits are for angioedema [7–9]. It is important to ensure that the therapies indicated for angioedema are appropriate and cost-effective.

Since 2015, icatibant has been introduced to the WRHA formulary in the form of three doses at Winnipeg Health Sciences Center (HSC): this is the first inclusion of this drug in a Canadian center’s formulary. This decision was driven by the conclusions of an RCT published in 2015 that showed icatibant to be effective in reducing the time to complete resolution of ACEIIAE [2]. However this result was challenged by two larger RCTs in 2017 that failed to replicate the benefits shown in the original 2015 trial [4, 6]. Therefore the evidence supporting the use of icatibant for ACEIIAE is controversial, along with its use in other angioedema tentatively hypothesized to arise from the bradykinin-mediated pathway or supported by small case series (e.g. tPA mediated, acquired and idiopathic angioedema) [10–12].

The price of icatibant 30 mg is approximately $4300 USD [13]. This high cost has necessitated that each dose be approved by the Allergist on call before it may be dispensed from the WRHA central pharmacy at Winnipeg Health Sciences Center. These considerations of cost and effectiveness indicate that the current use of icatibant within the WRHA’s facilities should be assessed.

The present study was conducted to ensure the current usage of icatibant is appropriate, timely, efficacious and targeted to the correct population. We explore if requests for icatibant are made in the context of appropriate patient evaluation, the timeline from patient presentation through initiation of therapy, if icatibant is efficacious in reducing angioedema per the defined endpoints of previous RCTs, and the characteristics that predispose patients to either an adequate or inadequate response to icatibant.

## Methods

We undertook a retrospective chart review to characterize the use and response to icatibant in the WRHA. A total of 21 charts were reviewed accounting for 23 administrations of icatibant in the WRHA dating from January 2014 to May 2019. One chart did not yield any information relevant to this study.

### Data extraction

The following was characterized from each chart: demographic history, investigations, medical history, angioedema history, family history, history of presenting illness, and treatment and evolution of the angioedema. Regarding icatibant’s usage, the following parameters were extracted: time of Allergist consult, time icatibant was dispensed by the pharmacy and the time of dose administration. Regarding each patient’s course, the following parameters were extracted: time to onset of symptom relief (OSR) and time to final clinical outcome (FCO; full resolution per physician or discharge, whichever first). Regarding investigations, the following parameters were recorded: complete blood count (CBC) with differential, electrolytes, chest x-ray, electrocardiogram (EKG), C4, and C1-estease inhibitor (C1-INH) level and function. Regarding the treatment of angioedema, the following treatments were recorded: antihistamines, glucocorticoids, epinephrine, C1-INH and fresh frozen plasma (FFP). Laboratory investigations were performed by WRHA laboratories. Atopy describes an umbrella of allergic diseases such as allergic rhinitis, asthma, eczema or food allergy. Symptoms of mast-cell activation were defined as symptoms of pruritic wheal and flare skin reactions, urticaria or other symptoms characteristic of histamine release.

### *Expected values (Table **2**)*

Per the Canadian Society of Allergy and Clinical Immunology’s 2014 guidelines, there is a lack of evidence on recommending specific treatments for HAE with normal C1-INH (HAE-nC1INH) [14]. We used these guidelines for treatment of HAE types I and II as reference to compare our patients with HAE with normal C1 esterase inhibitor and function. For the initial treatment or evaluation of patients, each n was constructed by excluding non-relevant patients from analysis. For instance, for “Pertinent negatives—ACEI:” n consists of the set of patients who could have had angiotensin-converting (ACEI) as a cause of angioedema (i.e. no prior explanatory diagnosis).

### Time to response data

To determine the overall time to response for patients with repeat presentations for the same condition the median duration was chosen as the representative value (e.g. Patient A with HAE-nC1INH who presented 3 times for angioedema and was treated with icatibant each time). The FAST-3 study was chosen for all survival analysis comparisons due to the alignment of their endpoint definitions with the endpoints we could extract [5]. For consistency, FAST-3 was also chosen to provide a comparator value for our patients with ACEIIAE to assess the performance of icatibant in ACEIIAE versus HAE-nC1INH. Non-laryngeal data from FAST-3 were used as comparators for this study’s HAE data, due to our high prevalence of non-laryngeal presentations. FAST-3’s laryngeal data were used to compare this study’s ACEIIAE data for similar reasons. Comparable endpoints between our study and Sinert et al.’s RCT led us to use their study to compare the performance of icatibant for ACEIIAE to ACEIIAE’s natural history [4]. While there were two other studies on the use of icatibant in ACEIIAE, concerns about the validity of the study population were raised regarding Baş et al.’s 2011 study and the trial by Straka et al. [6] did not describe endpoint data that could be used for comparison [4].

### Statistical analysis

Data was analyzed using R (version 3.6.1) [15] with the package “survival” (version 2.44–1.1) [16]. Sex was evaluated using a binomial test, assuming sexes would be equally distributed. Medians were reported due to right skew of data, consistent with previous observations [2, 17]. For patient proportions in each subgroup, p-values were calculated using Fisher’s Exact Test and are presented as descriptive statistics. The 2-Tailed Single Sample Sign Test was used to compare our data to FAST-3 targets due to the non-gaussian distribution of sample-mean differences.

Time-to-event results stratified by potential predictors (e.g. HAE-nC1INH vs. ACEI) were evaluated using a Peto–Peto Prentice method given non-proportionality of hazards; if no data was censored then the Mann–Whitney U-test was used. All applicable tests were 2-sided with an α of 0.05.

### Ethics

Ethics approval was obtained from the University of Manitoba Bannatyne Campus Research Ethics Board.

## Results

### Demographics, diagnoses and characterization of angioedema

We sought to characterize the presentations of angioedema requiring icatibant, knowing some patients would present more than once during the period of record eligibility due to the recurring nature of HAE and ACEIIAE. These data are found in Table 1. All HAE patients captured had HAE-nC1INH as their diagnosis and were significantly younger than non-HAE patients. Generally, the observed trend was that facial/oropharolaryngeal regions were mostly affected. HAE-nC1INH patients tended to have more abdominal symptoms compared to the non-HAE subgroup; the non-HAE subgroup seems to have more facial/oropharolaryngeal and cutaneous symptoms. Approximately half the presentations were for ACEIIAE; of these, 92% were first presentations without prior history of any angioedema. For the HAE-nC1INH subgroup, 5 patients accounted for 10 presentations. It seemed that there was an even spread of ACEI medications prescribed. For the rest of this paper, references to the present study population with “HAE” implicitly acknowledges that their specific diagnosis is HAE-nC1INH.

**Table 1:** Demographics, diagnoses and characterization of angioedema

Category	n	Stratification	Count (% of n)	p-value (*< 0.05)
Presentations with icatibant dispensation	23	NA	23 (100)	NA
Diagnosis	23	HAE nC1INH	10 (43) 10 (43)	
		ACEIIAE	11 (48)	
		Not yet diagnosed	1 (4)	
		tPA-induced angioedema	1 (4)	
Sex	23	Any diagnosis		
		Male	8 (35)	0.11
		Female	15 (65)	
	10	HAE		
		Male	3 (30)	0.95
		Female	7 (70)	
	13	Non-HAE		
		Male	5 (38)	0.87
		Female	8 (62)	
Age	23	All—median years (range)	57.5 (35–82)	NA
	10	HAE—median years (range)	52 (35–60)	0.02*
	13	Non-HAE—median years (range)	73.5 (37–82)	
Localization of angioedema	23	Any diagnosis		NA
		Face/oropharolarynx	16 (70)	
		Abdominal	9 (39)	
		Cutaneous	6 (26)	
	10	HAE		
		Face/oropharolarynx	4 (40)	
		Abdominal	8 (80)	
		Cutaneous	1 (10)	
	13	Non-HAE		
		Face/oropharolarynx	12 (92)	
		Abdominal	1 (8)	
		Cutaneous	5 (38)	
History of allergy	6	HAE	1 (17)	
		Non-HAE	5 (83)	
ACEI prescribed	11	Fosinopril	2 (18)	
		Lisinopril	3 (27)	
		Perindopril	3 (30)	
		Quinapril	1 (10)	
		Ramipril	2 (20)	

*NA* Not applicable

### Evaluation and treatment of patients prior to icatibant administration

We sought to describe the evaluation and treatment of patients prior to icatibant administration; the data is found in Table 2. All patients had a medication history elicited. Most patients had a comment on the presence or absence of ACEI usage documented in their chart. 65% of presenting patients were administered conventional medications before receiving icatibant, defined as antihistamines, glucocorticoids, epinephrine, C1-INH or FFP. Of those receiving prior conventional medication, the majority received antihistamines (60%) or corticosteroids (73%). Approximately half received epinephrine. In most presentations, C1-INH or FFP was administered. For patients who presented with a prior diagnosis of HAE-nC1INH, 30% received C1-INH prior to icatibant. No other treatments with conventional medications for angioedema were recorded for HAE-nC1INH patients. For patients without a prior diagnosis of HAE, two-thirds received antihistamines, 80% received glucocorticoids and just over half received epinephrine; only a minority received C1-INH or FFP.

**Table 2:** Evaluation and treatment of patients prior to icatibant administration

Category	n	Stratification	Count (%)	Expected (%)	p-value (*< 0.05)
Medication history taken	23	NA	23 (100)	NA	1.00
Pertinent positive or negative recorded	12	ACEI	10 (83)	100	0.23
		ARB	3 (25)		0.00*
		NSAID	5 (42)		0.00*
		Estrogen exposure	1 (8)		0.00*
		Family history	1 (8)		0.00*
		Allergies	11 (92)		0.50
Conventional medication administered before icatibant	15	Any diagnosis		NA	NA
		Antihistamines	9 (60)		
		Glucocorticoid	11 (73)		
		Epinephrine	7 (47)		
		C1-INH	4 (27)		
		FFP	5 (33)		
		HAE (n = 3)			
		Antihistamines	0 (0)		
		Glucocorticoid	0 (0)		
		Epinephrine	0 (0)		
		C1-INH	3 (20)		
		FFP	0 (0)		
		Non-HAE (n = 12)			
		Antihistamines	10 (67)		
		Glucocorticoid	12 (80)		
		Epinephrine	8 (53)		
		C1-INH	1 (7)		
		FFP	5 (33)		
Treatment if signs of mast cell activation	2	Epinephrine	2 (100)	100	1.00
		Antihistamines	2 (100)		1.00
		Glucocorticoids	2 (100)		1.00
Treatment if no signs of mast cell activation	21	Epinephrine	6 (29)	0	0.01*
		Antihistamines	8 (38)		0.00*
		Glucocorticoids	9 (43)		0.00*
CBC before icatibant	23	Any diagnosis	17 (74)	100	0.01*
	10	Prior HAE diagnosis	4 (40)		0.01*
	13	No HAE diagnosis	13 (100)		1.00
Basic chemistry	23	Any diagnosis	12 (52)	100	0.00*
	10	Prior HAE diagnosis	2 (20)		0.00*
	13	No HAE diagnosis	10 (77)		0.11
C4 level	23	Any diagnosis	9 (38)	100	0.00*
	10	Prior HAE diagnosis	4 (40)		0.01*
	13	No HAE diagnosis	5 (38)		0.00*
C1-INH level	23	Any diagnosis	11 (48)	100	0.00*
	10	Prior HAE diagnosis	5 (50)		0.02*
	13	No HAE diagnosis	6 (46)		0.00*
C1-INH function	23	Any diagnosis	7 (30)	100	0.00*
	10	Prior HAE diagnosis	4 (40)		0.01*
	13	No HAE diagnosis	3 (23)		0.00*

*NA* Not applicable

Less than 10% of patients had signs of mast cell activation, such as urticaria. These patients were all treated with epinephrine, antihistamines and glucocorticoids. Among those who did not have those signs, a significant non-zero number of them were treated with epinephrine, antihistamines or glucocorticoids.

If a patient did not have a prior explanatory HAE diagnosis, they were all evaluated with a CBC and most were also evaluated with a basic chemistry. However, less than half were evaluated with a C4 level, or C1-INH level or C1-INH function laboratory assay.

### Timeline to treatment, allergist consult, icatibant dispensation and administration

We sought to describe the treatment timeline in angioedema. The data is presented in Table 3. The median time from presentation to receiving conventional treatment was 1.29 h; from presentation to Allergist consult was 1.77 h; from presentation to icatibant dispensation by the central pharmacy was 2.40 h; and from presentation to icatibant administration was 2.95 h. Compared to patients who did not initially receive conventional treatment, those receiving conventional treatment had a median time to allergy consult of 4.15 h (vs. 1.15, p = 0.10), median time to pharmacy dispensation of 5.75 h (vs. 1.35, p = 0.03) and median time to icatibant administration of 7.78 h (vs. 2.06, p = 0.03).

**Table 3:** Timeline to treatment, Allergist consult, icatibant dispensation and administration

Event	Stratification	n	Median time (IQR), from ED presentation, hours	p-value (*< 0.05)	n	Median time (IQR), from last event, hours	p-value (*< 0.05)
ER presentation	NA	22	0	NA	NA	0	NA
Conventional treatment	NA	12	1.29 (0.25–3.89)		12	See left	
Allergist consult	Unstratified	22	1.77 (0.59–5.51)		N/A		
	Conventional^a^	11	4.15 (0.85–15.63)	0.10	11	2.42 (0.42–4.08)	
	Without^b^	11	1.15 (0.43–2.03)		11	See left	
Pharmacy dispensation	Unstratified	22	2.40 (0.87–6.15)	NA	22	0.40 (0.22–0.98)	
	Conventional^a^	11	5.75 (1.69–16.67)	0.03*	11	0.60 (0.23–1.60)	0.10
	Without^b^	11	1.35 (0.60–2.88)		11	0.35 (0.17–0.80)	
Icatibant administration	Unstratified	21^c^	2.95 (1.58–7.05)	NA	21^c^	0.55 (0.35–0.80)	NA
	Conventional^a^	10	7.78 (2.45–17.47)	0.03*	10	0.77 (0.55–0.82)	0.10
	Without^b^	11	2.06 (1.18–3.27)		11	0.38 (0.23–0.75)	

*NA* not applicable, *IQR* inter-quartile range

^a^Conventional = treated first with conventional treatment

^b^Without = no initial conventional treatment

^c^One patient was not administered icatibant due to symptom relief and was excluded from this analysis

### FCO and times to OSR and achievement of FCO

FCO was recorded for 91% of patient presentations (Table 4). The median time to first subjective report of OSR was calculated for all groups, then stratified by prior-HAE diagnosis, presumptive ACEIIAE, and whether there was facial/oropharyngeal involvement. These data are represented in Table 4. The pooled median time to OSR was significantly longer than reported in FAST-3. All strata other than ACEIIAE was not significantly different from FAST-3; the median time to OSR for ACEIIAE was 4.86 h compared to the reference of 0.8 h from FAST-3 (p = 0.01). The response to icatibant for HAE and ACEIIAE was compared to natural history references from Lumry et al. [5] and Sinert et al. [4], respectively. Icatibant-treated ACEIIAE’s median time to OSR was significantly longer than icatibant-treated HAE’s time and not significantly different from the median time to OSR in ACEIIAE’s natural history. Regarding median time to FCO, icatibant-treated HAE reached the endpoint significantly faster than HAE’s natural history would suggest. Icatibant-treated ACEIIAE’s median time to FCO was not significantly different from than what the diagnosis’s natural history would suggest. The pooled median time to achievement of FCO was not significantly different from the FAST-3 comparator of 8.0 h overall (6.0 h for facial/oropharolaryngeal involvement) [5].

**Table 4:** Final clinical outcome (FCO) and times to onset of symptom relief (OSR) and achievement of FCO

Category	n	Stratification	Hours (95% CI)	Reference value	p-value (*< 0.05)
FCO available	21	Any outcome	NA	NA	NA
	18	Fully resolved			
	3	Discharged without complete resolution			
Median time to OSR	21	All	1.34 (1.00–5.63)	0.8^b^	0.04*
	10	HAE vs natural history	1.13 (0.75–NE)	0.8^b^ 3.5^b^	0.34 0.10
	9^a^	ACEI-induced (n = 9) vs. natural history	4.86 (2.17–NE)	0.8^b^ 1.6^c^	0.01* 0.29
	14	Face/oropharolarynx symptoms present	2.17 (0.83–NE)	0.7^b^	0.09
	7	Face/oropharolarynx symptoms not present	1.00 (0.75–NE)	0.8^b^	0.45
Median time to FCO	21	All groups	7.75 (2.5–43.41)	8.0^b^	1.00
	10	HAE vs natural history	3.50 (1.83–NE)	8.0^b^ 36.0^b^	0.11 0.02*
	9^a^	ACEI-induced vs. natural history	19.53 (8.13–NE)	4.5^b^ 4.0^c^	0.18 0.18
	14	Face/oropharolarynx symptoms present	8.93 (4.67–73.28)	6.0^b^	0.42
	7	Face/oropharolarynx symptoms not present	2.18 (1.83–NE)	8.0^b^	0.45
Median time from attack onset to icatibant dose	10	HAE	9.58 (8.20–NE)	Pairwise	0.80
	9	ACEI-induced	16.53 (4.25–NE)		

*FCO*  Final clinical outcome, *OSR* onset of symptom relief, *NE* not estimable, *NA* not applicable

^a^Two presentations were for neither HAE or ACEIIAE

^b^From Lumry et al. [5]

^c^From Sinert et al. [4]

### Stratified analysis of possible confounding factors affecting response to icatibant

The data for median times to OSR and FCO were stratified into pairs of attributes and then compared as Kaplan–Meier estimates (Table 5). Presentations with HAE as a prior explanatory diagnosis had a shorter time to FCO: 3.50 vs 19.53 h (p = 0.02). Patients who were treated with glucocorticoids before icatibant had a significantly longer time to FCO (19.53 vs. 3.50 h; p = 0.03).

**Table 5:** Stratified analysis of possible confounding factors affecting response to icatibant

Category	Stratification	Time to OSR, median, hours	p-value (*< 0.05), pairwise	Time to FCO, median, hours	p-value (*< 0.05), pairwise
History or diagnosis	HAE ACEI-induced	1.13 4.86	0.05	3.50 19.53	0.02*
	Atopy None	8.35 7.94	0.40	19.53 7.94	0.40
	Smoking None	1.00 3.20	0.60	9.75 7.38	0.70
Body system	Face/oropharolarynx involvement None	2.17 1.00	0.50	8.93 2.50	0.10
	Abdominal involvement None	1.25 2.17	0.71	2.92 14.64	0.05
	Cutaneous involvement None	1.00 1.43	0.30	18.83 7.75	0.40
Initial treatment	Antihistamine None	3.13 1.13	0.40	14.63 4.08	0.08
	Epinephrine None	4.08 1.34	0.90	19.53 5.53	0.10
	Glucocorticoids None	4.08 1.00	0.20	19.53 3.50	0.03*
	C1-INH None	3.20 1.13	0.70	6.47 7.75	0.90
	FFP None	4.53 1.14	0.40	9.75 5.83	0.30

*FCO* final clinical outcome, *OSR* onset of symptom relief

## Discussion

The endpoints found in our study compared favourably to endpoints reported from FAST-3, an RCT that led to icatibant’s approval for treating HAE types I and II. Interestingly, our results support a potential role for icatibant in the treatment of HAE-nC1INH but not in ACEIIAE. Patients’ likelihood of responding to icatibant should be identified using medication review and treatment and family histories to facilitate timely treatment.

The overall time-to-outcome data from this study resembles the results published in FAST-3. The time to FCO achieved in this study was defined as the time to when icatibant-receiving patients were declared to have their angioedema resolved or were discharged, whichever was first. Comparisons were nonsignificant even when our data was stratified analogously to FAST-3’s laryngeal/nonlaryngeal arms or to compare FAST-3’s target indication (HAE types I and II) to our study population’s most prevalent off-label use (ACEIIAE). On one hand, this implies that our real-world experience may be consistent with RCT data studying a population with strict membership criteria. This supports the growing body of open-label trial evidence that icatibant may be effective in the treatment of HAE-nC1INH [18–21]. On the other hand, it is also true that in some cases our sample sizes may not have been powered enough to reliably detect a difference in effect.

The median time to OSR was greater in our data compared to FAST-3’s reported result (Table 4: 1.34 vs. 0.8, p = 0.04). This may have been for multiple reasons. FAST-3 was an RCT with a strict assessment schedule designed to identify various parameters, such as time to OSR. This contrasts with the routine procedure in a hospital, where patients may be assessed less regularly due to staff workload and that patients may not be subject to rigorous interviews assessing their symptoms’ evolution. In the FAST-3 trial, participants were assessed every 30 min for the first 4 h then at the 5-, 6-, 8- and 12-h time points [5]. Another potential reason is that FAST-3 strictly enrolled patients who were diagnosed with HAE type I or II, whereas our study’s HAE population had normal C1-INH. Alongside this, an approximately equal amount of presentations for angioedema were ACEI-associated without history of HAE. Finally, the comparator value from FAST-3 (0.8 h) was chosen because it represented the most robustly powered trial arm—nonlaryngeal angioedema—however, our initial analysis pooled patients with angioedema of any anatomic site.

We then stratified data by HAE-status, ACEI-induced, and facial/oropharolaryngeal-status to explore possible reasons for the difference in median time to OSR. “ACEI-induced” was a category rather than “Non-HAE-nC1INH” because the latter would have included a case of tPA-associated angioedema and a case of angioedema not yet diagnosed: these potentially have different etiologies from ACEIIAE. Stratifying the data also allowed comparison of FAST-3 laryngeal data with the subset of our population who had facial/oropharolaryngeal symptoms (p = 0.09: median time to OSR; p = 0.42: median time to FCO).

Analyzing these subgroups revealed that median time to OSR was significantly longer in ACEIIAE compared to the reported time in FAST-3 for HAE types I and II (p = 0.01). On its own, this suggests that ACEI-angioedema may not respond to icatibant as well, if at all, as HAE types I and II. Previous studies have led to an understanding that blocking the B2 receptor attenuates the hemodynamic effects of ACEI [22]. A study by Hubers et al. further elucidated the pathogenesis of ACEIIAE by supporting the hypothesis that decreased bradykinin degradation played a role in the pathogenesis of ACEIIAE [23]. This suggests that the negative results from 2 of 3 RCTs of icatibant treatment in ACEIIAE may be due to an extended delay between attack onset and icatibant treatment or that additional vasoactive compounds may underly the etiology.

To address this, we stratified the study group again into HAE-nC1INH and ACEIIAE groups and performed survival analysis on the interval between the time to attack onset and icatibant administration (Table 4). These curves were not significantly different: HAE-nC1INH’s median interval was 9.58 h vs ACEIIAE’s median interval of 16.53 h (p = 0.80). This suggested that our ACEI-induced subgroup may have had additional vasoactive compounds or pathways contributing to their angioedema etiology. However, these data were obtained retroactively from hospital records and in several cases the time of attack onset was found to be approximate and/or inconsistent. To further investigate, the icatibant-treated ACEIIAE group was compared to natural history endpoints reported by Sinert et al. [4]: our median times to OSR and FCO did not differ from placebo (p = 0.29 and 0.18, respectively). This supports the understanding that even though the B2 receptor may interact with ACEIs’ effects, this is not sufficient to be clinically relevant in the treatment of ACEIIAE. This is an important finding—the initial study which suggested icatibant to be effective for ACEIIAE may have influenced individuals to recommend it as a first-line treatment for ACEIIAE. However, this and other studies are now providing evidence against icatibant’s use for this indication [4, 6, 24].

Since the HAE-nC1INH subgroup’s median times to OSR and FCO were not different from FAST-3 results, this supports the use of icatibant for HAE-nC1INH patients. Icatibant has been approved by Health Canada for self-administration and is on the Manitoba Pharmacare formulary for HAE types I and II. Given the various steps involved (and time invested with each step) these three factors suggest an opportunity to explore expanding Manitoba Pharmacare criteria to include HAE-nC1INH. This will also reduce resources that are used when these patients present to an ED.

To investigate the other factors which may affect patients’ response to icatibant, we stratified our study population (Table 5). Comparing the Kaplan–Meier survival curves it was found that ACEIIAE had an almost sixfold (p = 0.02) longer time to FCO compared to HAE-nC1INH. While these two strata had significantly different times from each other, they were not significantly different from the reference value of 8.0 h.

Table 5 additionally indicates that treatment with glucocorticoids was significantly associated with an almost sixfold increase in median time to FCO. However, none of the HAE-nC1INH patients were administered glucocorticoids, so this comparison reduces to a comparison of the HAE-nC1INH and ACEIIAE groups’ responses to icatibant (Table 5, row 1).

Examining how patients are clinically evaluated, our data suggests that patients who receive icatibant are initially evaluated using predominantly interview rather than laboratory investigation. Patient evaluation norms seem sensitive to the common underlying reasons for a patient to present with angioedema without a previous corresponding diagnosis. Namely, importance is given to eliciting a general medication history, whether there is prior ACEI use or if there are any allergies. Notably, our findings imply that there is a lack of questioning on whether there is a family history of angioedema; this may be a consequence of charting practice rather than interview content. Those who present with a possible allergic etiology are treated appropriately.

Among patients who present without a prior explanatory diagnosis of angioedema, the previously mentioned etiologies that are less common seem to be neglected during history taking. Though angiotensin II receptor blockers (ARBs) and non-steroidal anti-inflammatory drugs (NSAIDs) have both been documented to be associated with angioedema, their absence as a pertinent negative was documented significantly less frequently than expected [25, 26]. This may be due to a combination of factors, such as that the relationship between ARB use and angioedema may be coincidental due to affected patients having recently used ACEIs [26]. The prevalence of NSAID allergic and pseudoallergic reactions is approximately 0.1–0.3%, partly reflecting the high exposure of the general population [27]. These reactions can be subdivided into six types—only one of which may present as angioedema without history of mast cell activation symptoms [25]. This suggests that the prevalence may be quite low and its consideration to be low yield if the patient seems to be improving and other, more probable, causes can be ascribed to the angioedema.

It was found that less than half of patients who present without a prior diagnosis received initial laboratory investigations, specifically C1-INH function and C1-INH and C4 levels. For patients presenting without a prior explanatory diagnosis, physicians should initiate investigations with the aim of identifying those who may respond to icatibant. Contributing to the lack of initial laboratory investigation is that it can take hours to days for these results to be reported, even if collected promptly. Future advances in laboratory medicine may expedite this, making bedside-stratification more accurate. However, these investigations should nonetheless be performed. Laboratory results may be useful in informing future management if those patients return to the ED with a recurrent attack and potentially decrease the time interval until effective treatment.

Future care of patients who present with angioedema without a prior explanatory diagnosis should consider limiting non-evidence-based treatment. Since icatibant’s effectiveness in treating non-HAE has recently been called into question by other studies and this present study [4, 6], greater emphasis should be placed on proper evaluation of patients to ensure patients receiving icatibant have relatively high pre-test probabilities of desired response. Interview content for ACEI-use and family history may be utilized to help predict which patients may respond to icatibant.

A future decision tool or algorithm may speed patients’ course in hospital. The median time from a patient’s arrival to the ED until receiving conventional treatment is 1.29 h and the time to when the Allergist is consulted is 1.77 h. The median time to the latter endpoint may be longer for those initially treated conventionally (4.15 h vs 1.15 h, p = 0.10). Overall, these intervals represent the time it takes for the patient to be initially assessed, treated, and for the Allergist to be involved. Though not presently detected, it is plausible a delay may arise if the ED independently trials conventional medications before consulting the Allergy service for further treatment options.

The difference between patients initially conventionally treated and patients who are not becomes significant when comparing time from ED presentation to pharmacy dispensation (5.75 vs. 1.35, p = 0.03) and from ED presentation to icatibant administration (7.78 vs. 2.06, p = 0.03). This implies that there is a substantial delay in receiving icatibant if a patient is first trialed on conventional treatment, possibly owing to the time required to prescribe and assess such treatment.

Though the time to Allergist consult is not significantly different between the groups receiving or foregoing conventional treatment, this may be due to our sample size. The time interval between the conventional medication trial and the Allergist consult is longer than the interval from presentation to Allergist consult in those without conventional treatment. This suggests that process factors affecting patients’ time course in hospital may not be sufficiently revealed by our data.

The median time from ED presentation to when the pharmacy dispenses icatibant is 2.40 h, with the time to receiving the dose at 2.95 h. The absolute difference between these medians of 0.55 h may be a lower estimate of the time delay between when icatibant is dispensed and when the dose is received. 21 of 22 dispensations occurred for patients who were geographically located in the same hospital (HSC) as the WRHA central pharmacy, which would be expected to decrease the time interval between these events. Insufficient data from other hospitals were available to determine summary statistics for comparison. Another contributing factor was that some patients may have had a relatively longer time interval until pharmacy dispensation but a shorter subsequent interval until icatibant administration. To examine this, the time between each icatibant dispensation and administration was calculated; the median time was found to be 0.55 h. This appears to be a reasonable representation of the time required for the dose to travel from the pharmacy to the appropriate destination, be received and then documented.

During the review of medical records, no adverse events related to the administration of icatibant were noted. Adverse event rates associated with icatibant have been reported in a range from 7 to 53% during the course of RCTs [2, 4–6, 28] The differences in reported range may stem from the different sample sizes and definitions or thresholds for classification of adverse events in these trials. That no adverse events were noted during the review of records could be due to the ED setting, where it would be expected that only significant adverse events would be documented. In contrast, clinical trial settings are expected to be more sensitive and watchful for adverse events. Our sample size is also relatively small (23 presentations) compared to the referenced trials, being larger than only two of the trials’ adverse event analysis arms (Baş et al.: n = 15; Straka et al.: n = 12) [2, 6]. Capturing adverse event rates with a sensitivity approaching that in clinical trials may require deliberate protocols to be enacted so that patients are explicitly assessed for such reactions.

Limitations of our study include a relatively small sample size and its retrospective nature. These factors have combined with the local healthcare system’s limited capacity for testing to prevent further fruitful differentiation of our patients’ HAE-nC1INH subtypes. Future directions would include recruiting a population of HAE-nC1INH patients who had their pathophysiology further characterized (e.g. FXII-HAE, PLG-HAE, etc.) to compare their responses to icatibant. A local study recently characterized a subset of the local HAE-nC1INH patients who had positive family histories but was similarly restricted by local resources [29]. Further study of patient outcomes should seek to control for the variable interval between the onset of a patient’s angioedema attack and when they present to the study center. Future retrospective chart reviews may benefit from a longer duration of data collection to further power the statistical analysis. Parallel initiatives may include piloting a decision tool to aid the biochemical investigation and management of patients presenting with angioedema.

## Conclusion

In summary, we sought to describe the experience of icatibant in the WRHA’s hospitals. Our experience implies that icatibant may be an effective treatment for HAE-nC1INH and contributes to a growing body of evidence from RCTs. Of patients receiving icatibant, approximately half had ACEIIAE, a diagnosis for which this study and others have called into question as an indication for icatibant. Particularly, stratification of time data into HAE and ACEIIAE groups showed that the median time to OSR was significantly greater in the latter group. The response of ACEIIAE to icatibant was similar to the natural history of ACEIIAE, suggesting non-B2 receptor mechanisms may contribute to its pathogenesis. The growing ambivalence for icatibant’s use in ACEIIAE may lead to changes in local guidelines and substantial cost-savings. Interview content for ACEI-use and family history should be used to stratify patients into groups of more- or less-likely responders to facilitate the timely treatment of patients with icatibant. Future advances in laboratory medicine and the pathophysiology of angioedema should be pursued with the goal of identifying effective bedside predictors of response to icatibant. Patients who are previous responders to icatibant, are diagnosed with HAE or are otherwise predicted to respond may be able to bypass the customary conventional medication trial, thereby decreasing time to icatibant administration. There exists an opportunity to prospectively study the delays in icatibant administration. Overall, our study encourages the further use of icatibant, however, possibly not for ACEIIAE.

## Data Availability

The datasets used and/or analyzed during the current study are available from the corresponding author on reasonable request.

## Abbreviations

ACEI: Angiotensin-converting enzyme inhibitor
ACEIIAE: Angiotensin-converting enzyme inhibitor induced angioedema
ARB: Angiotensin II receptor blockers
C1: Complement component 1
C1-INH: C1-estease inhibitor
C4: Complement component 4
CBC: Complete blood count
ED: Emergency Departments
EKG: Electrocardiogram
FAST-3: For Angioedema Subcutaneous Treatment-3
FCO: Final clinical outcome
FFP: Fresh frozen plasma
HAE: Hereditary angioedema
HAE-nC1INH: Hereditary angioedema with normal C1 esterase inhibitor and function
HSC: Health Sciences Center
NA: Not applicable
NE: Not estimable
NSAID: Non-steroidal anti-inflammatory drugs
OSR: Onset of symptom relief
RCT: Randomized controlled trial
tPA: Tissue plasminogen activator
USD: United States Dollars
WRHA: Winnipeg Regional Health Authority
